# The Gene Master Regulators (GMR) Approach Provides Legitimate Targets for Personalized, Time-Sensitive Cancer Gene Therapy

**DOI:** 10.3390/genes10080560

**Published:** 2019-07-25

**Authors:** Sanda Iacobas, Nneka Ede, Dumitru A. Iacobas

**Affiliations:** Personalized Genomics Laboratory, Center for Computational Systems Biology, Roy G. Perry College of Engineering, Prairie View A&M University, Prairie View, TX 77446, USA

**Keywords:** papillary thyroid cancer, BCPAP cells, 8505C cells, prostate cancer, LNCaP cells, DU145 cells, kidney cancer, HL-60 cells, cancer gene software

## Abstract

The dynamic and never exactly repeatable tumor transcriptomic profile of people affected by the same form of cancer requires a personalized and time-sensitive approach of the gene therapy. The Gene Master Regulators (GMRs) were defined as genes whose highly controlled expression by the homeostatic mechanisms commands the cell phenotype by modulating major functional pathways through expression correlation with their genes. The Gene Commanding Height (GCH), a measure that combines the expression control and expression correlation with all other genes, is used to establish the gene hierarchy in each cell phenotype. We developed the experimental protocol, the mathematical algorithm and the computer software to identify the GMRs from transcriptomic data in surgically removed tumors, biopsies or blood from cancer patients. The GMR approach is illustrated with applications to our microarray data on human kidney, thyroid and prostate cancer samples, and on thyroid, prostate and blood cancer cell lines. We proved experimentally that each patient has his/her own GMRs, that cancer nuclei and surrounding normal tissue are governed by different GMRs, and that manipulating the expression has larger consequences for genes with higher GCH. Therefore, we launch the hypothesis that silencing the GMR may selectively kill the cancer cells from a tissue.

## 1. Introduction

A very rich literature compared gene expression profiles in tissues collected from healthy and cancer donors to identify the transcriptomic signatures of various cancer phenotypes (e.g., references [1,2,3,4,5]) that are periodically organized in the atlas form (e.g., references [6,7]). Nanostring launched recently a panel claiming to categorize the disease heterogeneity using 32 biological signatures involving 770 genes across 23 key breast cancer pathways (https://www.nanostring.com/products/gene-expression-panels/gene-expression-panels-overview/ncounter-breast-cancer-360-panel). There are also available platforms to compare the gene expression profiles of surgically removed tumors with publicly available transcriptomes of cancer standard samples (e.g.: https://www.origene.com/products/tissues/tissuescan).

However, comparing samples collected from different persons may not be such a good idea, because in addition to the disease itself, the gene expressions depend on several other risk factors, making each human unique and with a unique life pathway. The never repeatable combination of factors affecting the gene expression profile is related to the person’s race, sex, age, genetic background, diet (affecting the microbiome), environment (exposure to ionizing radiation, carcinogenic toxins, stress), bad habits (smocking, drugs, alcohol), medial history, etc. Our gene expression studies on tissues from humans and animal models proved the transcriptomic profile dependence on strain and genetic background [8], sex [9], age [10], exposure to stress [11] and carcinogenic toxins [12], medical history and treatments [13]. This is why numerous investigators (e.g., reference [14] on papillary thyroid cancer) started to pair the transcriptomes of the cancer region with the cancer free adjacent tissue of the same patient.

The legitimate question in the transcriptomic signature quest is how many of the tested genes should be found as regulated (and how many of them up and how many down) to assess the designated form of cancer. Since there are 1.9 × 10^22^ distinct sets of genes if “only” 10 hits are needed from the 770 candidates of the nanostring nCounter^®^ breast cancer 360^TM^), there is no way to determine the predictive value of each of these sets from metadata. Moreover, in addition to the checked biomarkers, hundreds other genes are regulated and their (never repeatable) contributions to the cancer phenotype are neglected without knowing whether they are really negligible.

Still, let us suppose that a particular cancer form does have a transcriptomic signature, as was indicated by the meta-analysis of gene expression data from a large population of cancer patients. Are the signature genes valuable targets for the cancer gene therapy or are they good only for diagnosis (if the above supposition is true)? Being selected from the most frequently regulated genes in the population of cancer patients, the signature genes appeared as little protected by the cellular homeostatic mechanisms as are minor players. Therefore, restoring their right expression level may be of little consequence for the cell.

Instead of genes whose altered sequence or expression allegedly triggers a particular form of cancer in everybody [15,16,17,18,19,20], we proposed [21,22] that the most legitimate targets for cancer gene therapy are what we call “gene master regulators” (GMRs) of cancer nuclei. We defined the GMR as the gene whose highly protected expression level by the cellular homeostatic mechanisms sets up the cell phenotype by controlling major functional pathways through expression correlation with their genes. The high protection makes the GMR less sensitive to the environmental oscillations and therefore less variably expressed among biological replicas. However, small oscillations of the GMR expression are amplified by in-phase (positive) or anti-phase (negative) oscillations of the expression of many other genes. The composite metric termed Gene Commanding Height (GCH) was introduced by us [21,22] to establish the gene hierarchy in each cell phenotype, with the GMR having the top GCH.

The idea of “master regulators” was floating in genomics for a long time, most investigators looking for transcription factors whose regulation might have large downstream effects on the expression of many genes (e.g., references [23,24]). In addition to defining the GMRs in quantitative terms (by the GCH), our procedure does not restrict the GMR’s quest to transcription factors. Instead, we rank with respect to the GCH scores all coding AND non-coding RNAs whose abundance was adequately quantified via the used (RNA next generation sequence or microarray) platform. Moreover, in a recent paper [22], we have shown how five non-coding RNAs (ANKRD36BP2, FAM86B3P, H19, HCG11 and PMS2L2) regulate apoptosis in a surgically removed papillary thyroid cancer via expression correlation with apoptotic genes. Thus, our results are in line with other studies reporting the involvement of the non-coding RNAs in cancer development (e.g., references [25,26,27]) and therapy (e.g., references [28]).

The GMR approach is based on our Genomic Fabric Paradigm (e.g., references [29,30]) and runs on the computer software package CANCER-GMR (coded in Python v.3) with statistical and graphical user interface packages SciPy (https://www.scipy.org/) and Tkinter GUI. Our procedure can be applied to the four quarters of > 1 mm diameter cancer nucleus identified in a biopsy or surgically removed tumor, or to four dishes with the same cancer cell line.

The GMR targeting would be effective in selectively destroying the cancer cells from a tissue if: (1) cancer nuclei and surrounding quasi-normal tissues are governed by different GCH hierarchies, (2) expression manipulation of a gene has larger consequences in cells where that gene has higher GCH and (3) the GCH of the GMR is well above the GCHs of the next genes in the hierarchy. In this report, the GMR approach is illustrated with applications to our microarray data on human kidney, thyroid and prostate cancer samples, and on thyroid, prostate and blood cancer cell lines.

## 2. Materials and Methods

### 2.1. Tumor Samples

We have profiled the cancer nuclei (labeled CANCER1 and CANCER2, both Gleason score 4 + 5 = 9) and the surrounding normal tissues (NORM1 and NORM2) from the frozen, surgically removed prostate tumors of two men. The study was part of Dr. D.A. Iacobas’ project approved by the Institutional Review Boards (IRB) of the New York Medical College’s (NYMC) and Westchester Medical Center (WMC) Committees for Protection of Human Subjects. The approved IRB (L11,376 from 2 October 2015) granted access to frozen cancer specimens from the WMC Pathology Archives and depersonalized pathology reports, waiving patient’s informed consent. Four 2–8 mm 3 samples were collected from the cancer nuclei and normal tissues of each tumor. Although the selected regions were as homogeneous as possible, cells of different phenotypes were not completely eliminated, and expression of their genes affected the reported results.

In this report, we reprocess also our previously published microarray data from surgically removed, frozen preserved kidney (CCRCC—clear cell renal cell carcinoma) and papillary thyroid cancer tumors. Two primary cancer nuclei (labeled as PTA and PTB) and the cancer free (NOR) tissue from the right kidney, together with a chest metastasis (MET) were profiled from a 74 years old man with metastatic CCRCC, Fuhrman grade 3 [21]. The unilateral, single, papillary carcinoma (PAP-C), pathological stage pT3NOMx and the cancer free surrounding tissue were collected from a 33-year-old woman [22].

No treatment was undertaken by the patients whose tumors were profiled and no result of our investigation was revealed to them or to their doctors to influence the therapeutic procedures. The purpose of the study was only to test the GMR approach and show the research community that there are other potentially valuable avenues in cancer therapy that deserve to be explored.

### 2.2. Cell Lines

The results for the surgically removed thyroid and prostate tumors were compared with those obtained from the commercially available standard human cancer cell lines: BCPAP, 8505C, LNCaP and DU145. We have also determined the GCH hierarchy in the human leukemia cell line HL-60. The HL-60 cell line was originally obtained at MD Anderson Cancer Center from a 36-year-old woman with acute promyelocytic leukemia [31].

The BCPAP cell line is a papillary thyroid carcinoma cell line isolated from a female patient, with a TP53 mutation in the codon 278 in heterozygosity (Pro→Leu) [32]. The 8505C cell line was established from undifferentiated thyroid carcinomas of a 78-year-old-female patient. Her tumor also contained residual well differentiated components, suggesting “well differentiated to undifferentiated carcinoma progression” [33]. We used both BCPAP and 8505C cell lines to test whether manipulation of the expression of a gene has larger transcriptomic consequences if that gene has a higher GCH. As mentioned in a previous publication [22], both cell lines were certified by the Genomic Facility of the Albert Einstein College of Medicine (Bonx, NY 10461, USA) (https://www.einstein.yu.edu/research/shared-facilities/cores/46/genomics/).

The LNCaP cells (Lymph Node Carcinoma of the Prostate) are androgen-sensitive adherent epithelial cells, obtained from a 50-year-old white male in 1977 [34]. The DU145 hormone insensitive cells were derived in 1976 from prostate adenocarcinoma metastatic to the brain of a 70-year-old white male [35].

### 2.3. Biological Replicas

The biological replicas (the quarters of a quadrisected homogeneous region of a tumor or four cell culture dishes of a cell line) can be considered as being the same system but subjected to slightly different environmental conditions. As such, the transcriptomic data provide valuable information on how much the genes resist or adapt to the external influences and how the variations of their expression levels are correlated to optimize the functional pathways. From the expression values in biological replicas we derive three independent measures to be used in subsequent analyses for each coding and non-coding RNA: (1) average level, (2) coefficient of variation and (3) expression correlation with each other RNA. The average expression level is used to identify what gene is up/down-regulated when compared two conditions. The coefficient of variation (CV) is used to estimate the control of the transcript abundance in each condition, and the expression correlation to identify and quantify the transcriptomic networks.

### 2.4. Microarray

We have used our standard protocol [36] for RNA extraction, purification, reverse transcription and fluorescent labeling, and hybridization with Agilent human 4 × 44 k gene expression two-color G2519F microarrays. (Full experimental details can be found in the genomic datasets cited at Section 3.1 below, deposited by us in https://www.ncbi.nlm.nih.gov/gds/?term=iacobas). The chips were scanned with an Agilent G2539A dual laser scanner and primary analysis performed with (Agilent) feature extraction v.12.0 software. All Agilent products (equipment, consumables and software were purchased from Agilent Technologies, Santa Clara, CA 95051, USA (https://www.agilent.com/cs/agilent/en/contact-us/united-states) All control spots as well as spots with corrupted or saturated pixels, or with forward fluorescence less than twice the background one in any of the four profiled biological replicas were removed from the analysis of that type of samples. 

### 2.5. Relative Expression Variation (REV) and Relative Expression Stability (RES)

All microarray platforms probe transcripts redundantly by several (unfortunately not uniform numbers of) spots. Therefore, instead of the coefficient of variation for the expression level in biological replicas as determined by one spot we use the Relative Expression Variation (REV, [37]) that takes into account all spots *R_i_* probing redundantly the same transcript *i*. REV is the mid-interval chi-square estimate of the pooled expression level CVs in biological replicates of that condition (cancer or normal) with a pre-established probability *ε* and number of degrees of freedom derived from the number of spots probing the same transcript:

(1) $$REV_{i}^{\left(condition\right)}(\varepsilon )=\frac{1}{2}\left(\sqrt{\frac{r_{i}}{\chi^{2}\left(r_{i};1-\varepsilon /2\right)}}+\sqrt{\frac{r_{i}}{\chi^{2}\left(r_{i};\varepsilon /2\right)}}\right)\sqrt{\frac{1}{R_{i}}\sum_{k=1}^{R_{i}}{\left(\frac{s_{ik}^{\left(condition\right)}}{\mu_{ik}^{\left(condition\right)}}\right)}^{2}}$$

where: *s_ki_* and *μ_ki_* are respectively the standard deviation and the average expression level of the gene *i* probed by spot *k*, *r_i_* = *λR_i_*−1 is the number of degrees of freedom, *λ* (≥4) is the number of biological replicas and *R_i_* is the number of spots probibng redundantly transcript *i.*

In our experiments with Agilent 4 × 44 k microarrays the number of spots probing redundantly the same transcript ranges from 1 to 28, so that *r* = 3, 4, …, 71. Our Cancer GMR software (presented in Section 2.10 below) has uploaded the chi-square values for all these values of r and for *ε* = 0.010, 0.025, 0.050, 0.100, values less than 0.010 being useless because of the technical noise affecting the gene expression levels. However, in the applications presented here, we used *λ* = 4 and *ε* = 0.05 for which the correction coefficient of the CV ranges from 1.566 (*R* = 1) to 0.960 (*R* = 28). Therefore, *λ* and *ε* will be omitted from the next equations.

Relative Expression Stability (RES) is a measure we introduce to rank the priorities of the cell homeostatic mechanisms in controlling the right abundance of a particular transcript. RES is applied to all transcripts regardless of them translating into proteins or having only regulatory roles for the expression of other transcripts.

(2) $$RES_{i}=\ln\left(\frac{\langle REV\rangle }{REV_{i}}\right),\;\langle REV\rangle =\mathrm{median}\;\mathrm{REV}\;\mathrm{for}\;\mathrm{all}\;\mathrm{transcripts}$$

The log form was selected to assign positive values to the more stably expressed and negative ones to the less stably expressed genes than the median one.

### 2.6. Expression Regulation

Instead of an arbitrarily introduced (e.g., 1.5× or 2.0×) absolute fold-change, we consider a gene as significantly regulated in cancer with respect to the normal counterpart if the absolute expression ratio |*x*| exceeds the cut-off calculated for that gene.

(3) $$\begin{array}{l} \left|x_{i}\right|>CUT_{i}=1+\sqrt{2\left({\left(REV_{i}^{\left(cancer\right)}\right)}^{2}+{\left(REV_{i}^{\left(normal\right)}\right)}^{2}\right)},\;where:\\ \left|x_{i}\right|=\{\begin{array}{cc} \frac{\mu_{i}^{\left(cancer\right)}}{\mu_{i}^{\left(normal\right)}} & ,\;\mathrm{if}\;\mu_{i}^{\left(cancer\right)}>\mu_{i}^{\left(normal\right)}\\ -\frac{\mu_{i}^{\left(normal\right)}}{\mu_{i}^{\left(cancer\right)}} & ,\;\mathrm{if}\;\mu_{i}^{\left(cancer\right)}<\mu_{i}^{\left(normal\right)} \end{array},\;with:\;\{\begin{array}{c} \mu_{i}^{\left(cancer\right)}=\bar{\mu_{ik}^{\left(cancer\right)}}\\ \mu_{i}^{\left(normal\right)}=\bar{\mu_{ik}^{\left(normal\right)}} \end{array} \end{array}$$

*CUT* observes the uncertainty about expression regulation by taking into account the contributions of both biological variability and technical noise. CUT is not uniform among the quantified transcripts and takes >1 values that may be even smaller than 1.5.

However, rather than the popular percentage of the regulated out of quantified genes, we measure the change in the transcriptional profiles by the Weighted Pathway Regulation (WPR). WPR is not restricted to the regulated but ponders all quantified genes. We have previously used WPR to quantify the remodeling and recovery of functional genomic fabrics in heart [38], hypothalamus [13] and hippocampus [39]:

(4) $$\begin{array}{l} WPR\equiv \langle \mu_{i}^{\left(normal\right)}\left(\left|x_{i}^{\left(cancer\right)}-1\right|\right)\left(1-p_{i}^{\left(cancer\right)}\right)\rangle ,\;where:\\ p_{i}^{\left(cancer\right)}=\;p\text{-}\mathrm{val}\;\mathrm{of}\;\mathrm{the}\;\mathrm{heteroscedastic}\;t\text{-}\mathrm{test}\;\mathrm{of}\;\mathrm{the}\;\mathrm{equlity}\;\mathrm{of}\;\mathrm{the}\;\mathrm{mean}\;\mathrm{expressions} \end{array}$$

The percentage of the regulated genes regards all regulated genes as equal contributors regardless of the fold-change and *p*-value. In contrast, WPR weights the genes by considering the expression ratio (*x*), the *p*-value of the t-test of equal expression, and the average expression level in the normal tissue (*µ*^(*normal*)^).

### 2.7. Expression Correlation

Pearson pair-wise correlation coefficient *ρ_ij_* was computed for the log_2_ expressions of all *N(N − 1)/2* pairs that can be formed with the N adequately quantified distinct transcripts in all biological replicas. The correlation coefficient takes values from −1 to +1. Close to positive and negative unit *ρ*’s indicate that the expression of one gene of the pair has strong synergistic or antagonistic consequences on the expression of the other, without specifying what gene comes first. Expressions of synergistic partners fluctuate in phase, those of antagonistic partners fluctuate in antiphase. We believe that strong synergism and antagonism occur when the expressing genes are linked in a functional pathway, providing the “transcriptomic stoichiometry” that rules the expression levels of the involved proteins [40]. Close to zero correlation coefficient means either that the two genes are independently expressed (not networked in any functional pathway) or (very unlikely) that their synergistic correlation in some pathways is balanced by their antagonistic correlation in other pathways.

### 2.8. Gene Commanding Height (GCH)

The Gene Commanding Height (GCH) was introduced by us [21,22] to quantify the importance of each gene for the cell phenotype. We consider that the expression level of a critical gene for the cell phenotype should be under a stricter control/protection of the homeostatic mechanisms and therefore should have higher expression stability among biological replicas. The same gene should also have a major regulatory role by coordinating the expression of many other genes. If we are allowed a comparison, the most protected persons in the UK are the Queen and the Prime Minister. However, the Prime Minister is the Master Regulator but the Queen is not, owing to the power of the office to oversee all major sectors in the UK policy and economy.

(5) $$\begin{array}{l} GCH_{i}=\exp\left(RES_{i}+\underset{\mathrm{relative}\;\mathrm{coordination}\;\mathrm{power}}{\underset{︸}{\frac{\frac{1}{N-1}\sum_{j=1,j\neq i}^{N}\rho_{ij}^{2}}{\frac{1}{N(N-1)}\sum_{k=1}^{N}\left(\sum_{j=1,j\neq k}^{N}\rho_{kj}^{2}\right)}}}\right)≃\frac{\langle REV\rangle }{REV_{i}}\times \exp\left(4{\bar{\rho_{ij}^{2}}|}_{\forall j\neq i}\right) \end{array}$$

### 2.9. Gene Ontology and Functional Pathways

Whenever available through Gene Ontology Consortium [41,42], (www.geneontology.org) and/or Kyoto Encyclopedia of Genes and Genomes (KEGG, [43], (http://www.genome.jp)) functional pathways are assigned to the genes. This can be done either by using the KEGG Ontology number as in GAEV [44], (https://github.com/UtaDaphniaLab/Gene_Annotation_Easy_Viewer), either by the gene symbol as in our GMR-Pathway (see below).

In this report we considered the following KEGG pathways: APO (map hsa4210 apoptosis), BTF (hsa03022 basal transcription factors), CCY (hsa04110 cell cycle), CSP (hsa04062 chemokine signaling), OXP (hsa00190 oxidative phosphorylation), RCC (hsa05211 renal cell carcinoma) and RPO (hsa03020 RNA polymerase).

### 2.10. CANCER-GMR Software

Programs of our CANCER-GMR software package were designed using the Anaconda distribution of Python 3 with statistical and graphical user interface packages such as SciPy (https://www.scipy.org/) and Tkinter GUI (Graphical User Interface). The GMR Software Package includes executable programs to determine the absolute fold-change cut-offs (**CUT**) when comparing gene expression average levels in cancer and healthy tissues, identify functional pathways within KEGG dbase (**PATHWAY**), the Weighted Pathway Regulation (**WPR**), the Pearson correlation coefficients between the expression levels of all gene pairs (**CORRELATION**) and the Gene Commanding Height (**GCH**).

 

**#CUT#**

REV1 = CORRECTION * std1/mean1

REVV2 = CORRECTION * std2/mean2

CUT = 1 + np.sqrt(2 * (np.square(df[“REV1”]) + np.square(df[“REV2”])))

 

**#PATHWAY#**

gene = REST.kegg_get(species+’:’+gene).read()

### find all pathways in KEGG

if current_section == “PATHWAY”:

gene_identifiers = line[1 2:].split(“;”) #Splits each line based on ‘;’

 

**#WPR#**

avg_gch = avg_gch/length #avg of GCH of the genes in a certain pathway

wpr = np.mean(avg_gch * (abs(fc)-1) * (1-p_val))

 

**#CORRELATION#**

df = pd.read_csv(df, header = 0, na_values = “NaN”)

data = pd.concat([df[condition_input+”1”],df[condition_input+”2”],

df[condition_input+”3”], df[condition_input+”4”]], axis = 1)

logvalues = np.log2(data)

results = (logvalues.T).corr(method = ‘pearson’) #transpose and find correlation

pearsons_df = pd.DataFrame(results.values, columns = df[‘GeneName’], index = 

df[GeneName’].values) ## change to dataframe

 

**#GENE COMMANDING HEIGHT#**

expcon = median/cov #expression control

results = (logvalues.T).corr(method = ’pearson’) #transpose and find correlation

thesum = (results * results).sum(axis = 0)

### gene commanding height calculations

controlcoor = np.exp((4*thesum-1)/len(pearsons_df.index)-1)

gch = expcon.values * np.exp((4*thesum-1)/len(pearsons_df.index)-1).values

gch = pd.DataFrame(gch, dtype = ’float’)

### 2.11. Experimental Design to Validate the GMR Theory

**Figure 1:**
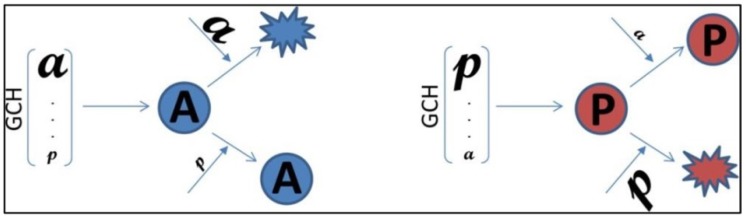
Experimental design to validate the Gene Master Regulators (GMR) theory. **a** is a gene with higher Gene Commanding Height (*GCH*) in the **A**-cells (here the 8505C anaplastic thyroid cancer cell line) than in the **P**-cells (here the BCPAP papillary thyroid cancer cell line), while **p** is a gene with higher *GCH* in the **P**-cells than in the **A**-cells. The theory is verified if transfection of a will induce significantly larger Weighted Pathway Regulation (*WPR*) in the **A**-cells than in the **P**-cells and transfection of **p** will induce larger WPR in the **P**-calls than in the **A**-cells

One way to validate the GMR theory is to establish the GCH hierarchy in two cell lines, transfect each line with genes having same expression level but different GCHs and compare the transcriptomic alterations (Figure 1). The theory is validated if the alterations of the same gene transfection are higher in the cells where that gene has a higher GCH. We tested the usefulness of the GMR approach for cancer gene therapy by stable lentiviral transfecting the human BCPAP (papillary) and 8505C (anaplastic) thyroid cancer cell lines with four genes *NEMP1*, *PANK2*, *DDX19B* and *UBALD1*. The selected genes have similar average expression levels (AVE, normalized to the median of all quantified transcripts) but significantly different GCH scores in the two cell lines.

## 3. Results

### 3.1. Experimental Data

The GMR approach is illustrated here with applications to our gene expression datasets from surgically removed human (kidney, thyroid, prostate) cancer tissues and commercially available human cancer cell lines deposited by us in the Gene Expression Omnibus of the National Center for Biotechnology Information (www.ncbi.nlm.nih.gov/gds). Data used in this report were from GSE72304 (a case of metastatic clear cell renal cell carcinoma (CCRCC), GSE97001 (a case of papillary thyroid cancer), GSE97002 (BCPAP papillary and 8505C anaplastic thyroid cancer cell lines) and from two cases of prostate cancer (GSE133891, GSE133906). Expression data for the GMR Theory validation on thyroid cancer cell lines BCPAP and 8505C were collected from GSE97031 (transfection with NEMP1), GSE97028 (DDX19B), GSE97030 (PANK2) and GSE97427 (UBALD1). Other expression data from cancer cell lines were collected from GSE72333 (DU145), GSE72414 (LNCaP) and GSE72415 (HL-60).

### 3.2. Expression Stability, Expression Correlation and Weighted Pathway Regulation

**Figure 2:**
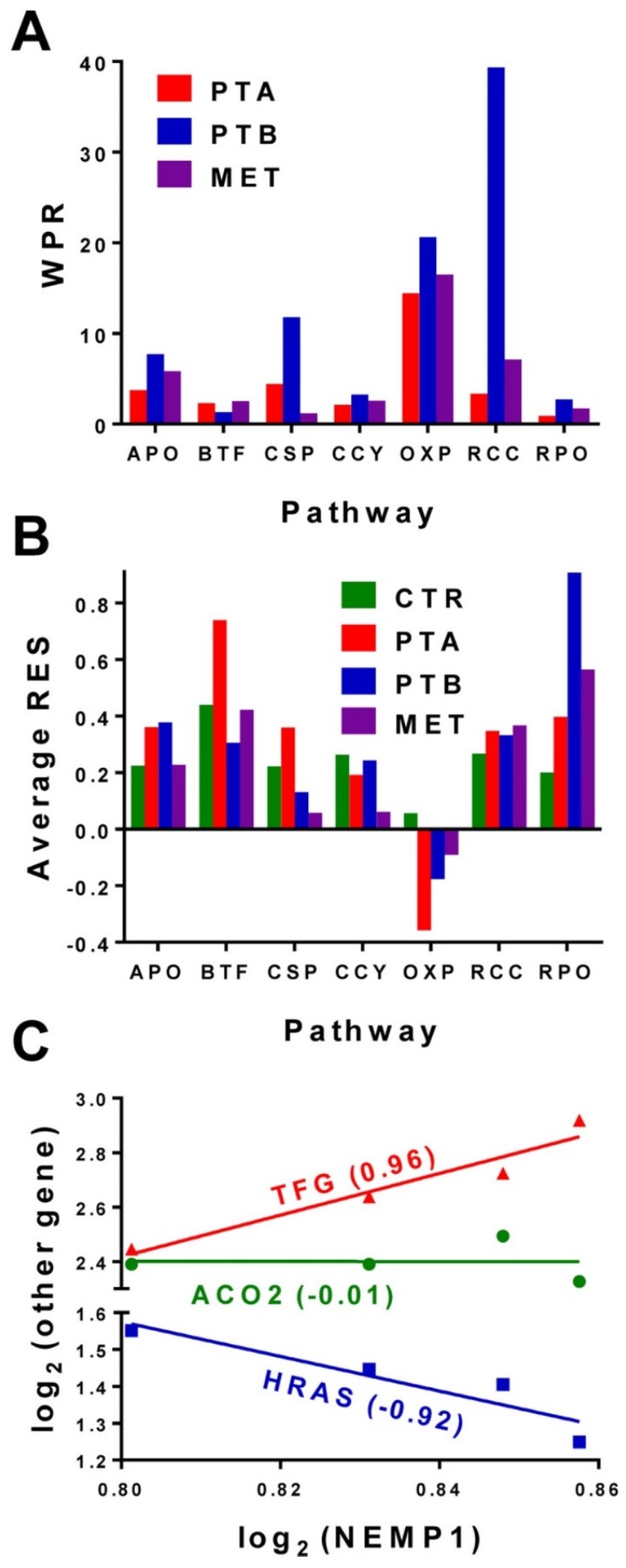
Expression data from [21]: (**A**). Average Relative Expression Stability (RES) of several functional pathways in the four regions profiled from the clear cell renal cell (CCRCC) samples. Note that the RNA polymerase (RPO) and basal transcription factors (BTF) are the most protected pathways, while the control of the oxidative phosphorylation (OXP) is relaxed, presumably to allow the cells to adapt the environmental conditions. (**B**). Weighted Pathway Regulation (WPR) analysis of several functional pathways. Pathways: APO = apoptosis, BTF = basal transcription factors, CCY = cell cycle, CSP = chemokine signaling, OXP = oxidative phosphorylation, RCC = renal cell carcinoma, RPO = RNA polymerase. (**C**). Example of the correlation analysis in BCPAP cells (expression data from [22]). *NEMP1* is synergistically expressed with *TFG* (trafficking from ER to golgi regulator), antagonistically expressed with *HRAS* (Harvey rat sarcoma viral oncogene homolog) and independently expressed with *ACO2* (aconitase 2, mitochondrial) in BCPAP cells.

The Relative Expression Stability (RES) can be used not only to establish the hierarchy of individual genes but also the hierarchy of functional pathways. Figure 2A presents the average RES scores of several pathways analyzed in each of the four regions profiled from the CCRCC samples. The averages were determined for 107 APO, 37 BTF, 131 CSP, 91 CCY, 100 OXP, 54 RCC and 31 RPO genes out of the 12610 distinct genes whose expression was adequately quantified in all four regions of the CCRCC samples. WPR analysis of the CCRCC samples for the same pathways returned the results from Figure 2B. Note that (by far) the most affected pathway was (as expected) RCC (the renal cell carcinoma) followed by OXP (oxidative phosphorylation).

Figure 2C illustrates the correlation analysis with examples of synergistically, antagonistically and independently expressed partners of *NEMP1* (nuclear envelope integral membrane protein 1) in the BCPAP cells. In our opinion, the strong correlations of the (not yet assigned to a pathway) *NEMP1* gene with the oncogenes *TFG* and *HRAS* indicate its potential role in the papillary thyroid cancer. *HRAS* is among the most documented genes whose mutations have been associated with thyroid cancer [45]. *TFG* gene is described in https://www.ncbi.nlm.nih.gov/gene/10342 as partially encoding several fusion oncoproteins and participating in several “oncogenic rearrangements resulting in anaplastic lymphoma and mixoid chondrosarcoma”. *TFG-MET* (MET proto-oncogene receptor tyrosine-kinase) translocation was reported in a follicular variant of the papillary thyroid carcinoma [46]. Interestingly, *NEMP1* was recently shown as promoting tamoxifen resistance in breast cancer cells [47]. Thus, the correlation analysis may be used to refine the maps of the functional pathways by determining the gene pairs whose correlated expression may result from a functional relationship between their encoded proteins.

### 3.3. Cancer Nuclei and Surrounding Normal Tissue Are Governed by Distinct GMRs

Table 1, Table 2 and Table 3 present the GCH scores of the top 3 genes in cancer nuclei and surrounding normal tissue in a case of metastatic clear cell renal cell carcinoma, a case of papillary thyroid cancer and two cases of prostate cancer.

In Table 1, only *ALG13* is an actionable GMR (for MET region) owing to its significantly higher GCH (82.95) with respect to the second gene, *NUDT18* (GCH = 48.40). Interestingly, *ALG13* (an early target of miR-34a) was reported as correlated with worse clinical outcomes for neuroblastoma [48]. Results in Table 1 also indicate that distinct cancer nuclei of the same tumor (PTA, PTB) may have a distinct gene hierarchy that explains their phenotypic diversity. Note also that *MIR1915HG*, previous names: chromosome 10 open reading frame 114, cancer susceptibility candidate 10 and cancer susceptibility 10, is a long non-coding RNA.

In a previous publication [22], we have presented the GCH scores of 78 cancer biomarkers, 44 oncogenes, 55 apoptosis genes and 120 ncRNAs in the cancer and normal areas of a surgically removed papillary thyroid tumor. In that selection of 297 genes, all but *RAB15* (GCH^(cancer)^ = 26.14) had GCH scores below 20. The complete GCH analysis of the expression data in the same tumor sample revealed that the GMR of the cancer area is *SPINT2* (GCH^(cancer)^ = 54.97). Interestingly, SPINT2 a transmembrane protein that inhibits serine proteases implicated in cancer progression [49], acts as a putative tumor suppressor when hypermethylated [50].

It is notable that *WFDC3*, the far above GMR of the androgen-sensitive prostate cancer LNCaP cells, was also reported as one of the most down-regulated gene in the ventral prostate of aged (18 moths) estrogen receptor β^−/−^ mouse [51]. Interestingly, the GMR of the cancer nucleus of the second man (*LOC145474*) is a non-coding RNA, confirming that both coding and non-coding RNAs may play dominant roles in prostate tumorigenesis [52]. To our knowledge, this is the first time that *LOC145474* is reported as related to a prostate cancer.

### 3.4. Experimental Validation of the GMR Theory

**Figure 3:**
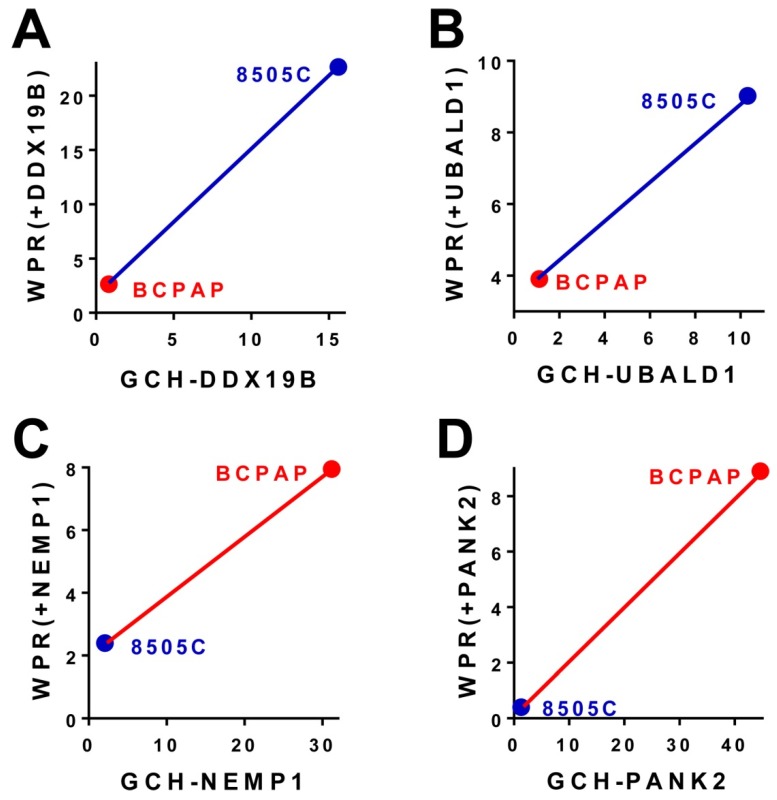
Data from GSE97031, GSE97028, GSE97030, GSE97427, [22]: Validation of the GMR Theory. (**A**,**B**). Stable transfection of genes with higher GCH in the 8505C cells than in BCAP cells had larger transcriptomic effects in 8505C cells as measured by the Weighted Pathway Regulation (WPR). (**C**,**D**). Stable transfection of genes with higher GCH in the BCPAP cells than in 8505C cells had larger transcriptomic effects in BCPAP cells as measured by the Weighted Pathway Regulation (WPR).

Our experimental results (summarized in Figure 3) on the 8505C (anaplastic, 3A & 3C) and BCPAP (papillary, 3B & 3D) human thyroid cancer cell lines stably transfected with *DDX19B*, *NEMP1*, *PANK2* or *UBALD1* (characteristics in Figure 1) indicate that:

(6) $$\begin{array}{lllllll} GCH_{NEMP1}^{\left(BCPAP\right)} & > & GCH_{NEMP1}^{\left(8505C\right)} & \Rightarrow & WPR_{NEMP1}^{\left(BCPAP\right)} & > & WPR_{NEMP1}^{\left(8505C\right)}\\ GCH_{PANK2}^{\left(BCPAP\right)} & > & GCH_{PANK2}^{\left(8505C\right)} & \Rightarrow & WPR_{PANK2}^{\left(BCPAP\right)} & > & WPR_{PANK2}^{\left(8505C\right)}\\ GCH_{DDX19B}^{\left(BCPAP\right)} & < & GCH_{DDX19B}^{\left(8505C\right)} & \Rightarrow & WPR_{DDX19B}^{\left(BCPAP\right)} & < & WPR_{DDX19B}^{\left(8505C\right)}\\ GCH_{UBALD1}^{\left(BCPAP\right)} & < & GCH_{UBALD1}^{\left(8505C\right)} & \Rightarrow & WPR_{UBALD1}^{\left(BCPAP\right)} & < & WPR_{UBALD1}^{\left(8505C\right)} \end{array}$$

We have also observed that transfections of NEMP1 and PANK2 significantly slowed down multiplication of BCPAP cells, while transfection of DDX19B or UBALD1 had little effect on these cells. By contrast, both transfections of DDX19B and UBALD1 significantly slowed down grow of 8505C cells, while transfection of NEMP1 and PANK2 had little effect. Together, these observations confirm that **expression manipulation of a gene has larger consequences in cells where that gene has higher GCH.**

### 3.5. Predicted Transcriptomic Alteration by GMR Manipulation

**Figure 4:**
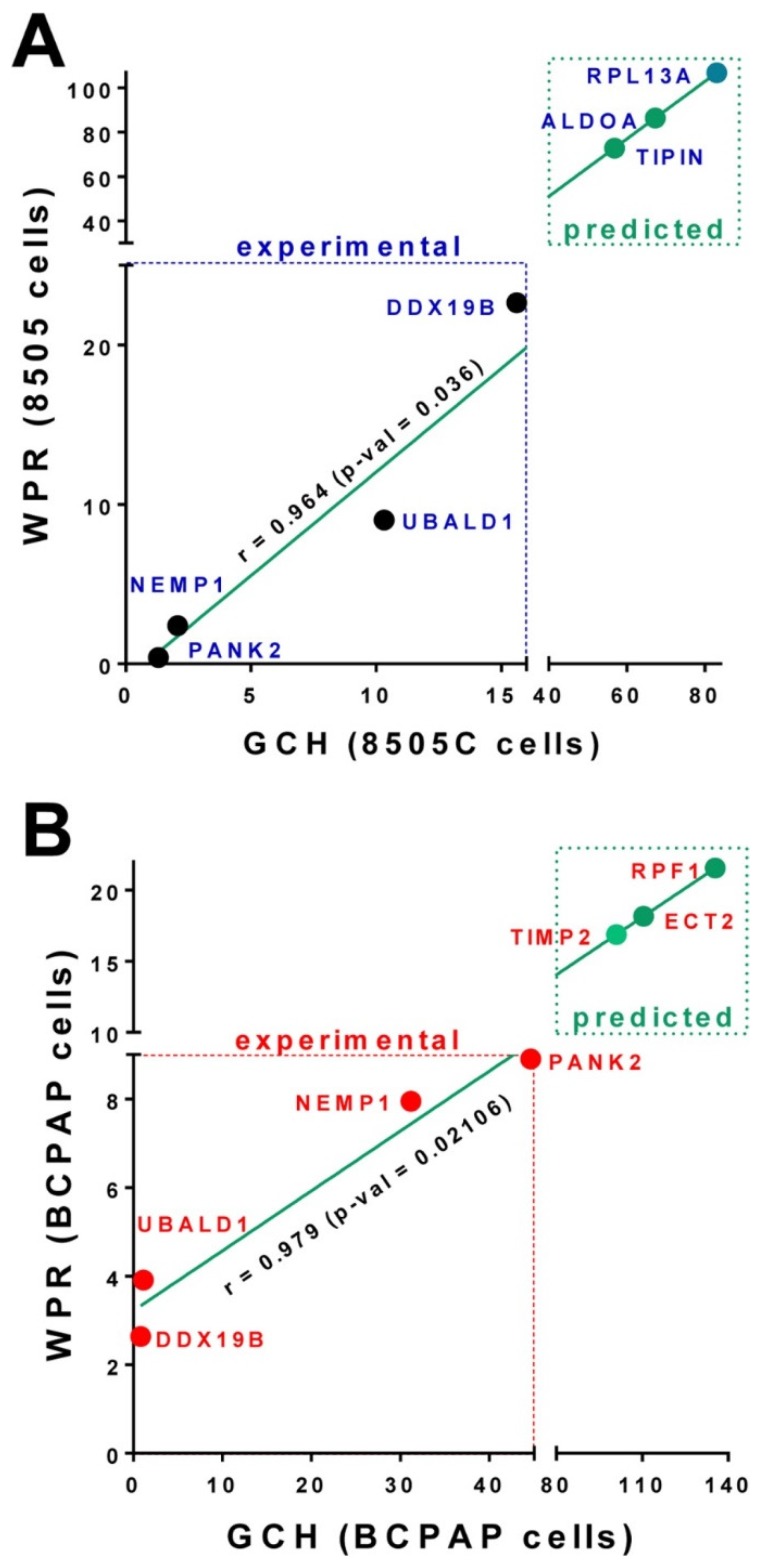
(**A**). Experimentally measured and theoretically predicted effects of stably transfecting *PANK2*, *NEMP1*, *UBALD1*, *DDX19B* and the top three genes (*RPL13A*, *ALDOA* and *TIPIN*) in the 8505C. (**B**). Experimentally measured and theoretically predicted effects of stably transfecting *PANK2*, *NEMP1*, *UBALD1*, *DDX19B* and the top three genes (*RPF1*, *ECT2* and *TIMP2*) in the BCPAP cells.

At the time, we had no possibility to alter the expression of the GMRs identified in the thyroid cancer cell lines 8505C and BCPAP. Figure 4 presents the predicted Weighted Pathway Regulation if significantly altering the expressions of the top three genes in the 8505C and BCPAP cells.

Interestingly, the GMR of the anaplastic cell line (*RPL13A*) was found as the most stably expressed gene in two ovarian cancer cell lines (UACC–1598 and SKOV3) subjected to two widely used anticancer treatment [53], confirming the major role played by this gene in stabilizing the cancer phenotype.

### 3.6. Ribosomal Genes Top the Hierarchy in the Acute Promyelocytic Leukemia HL-60 Cell Line

**Figure 5:**
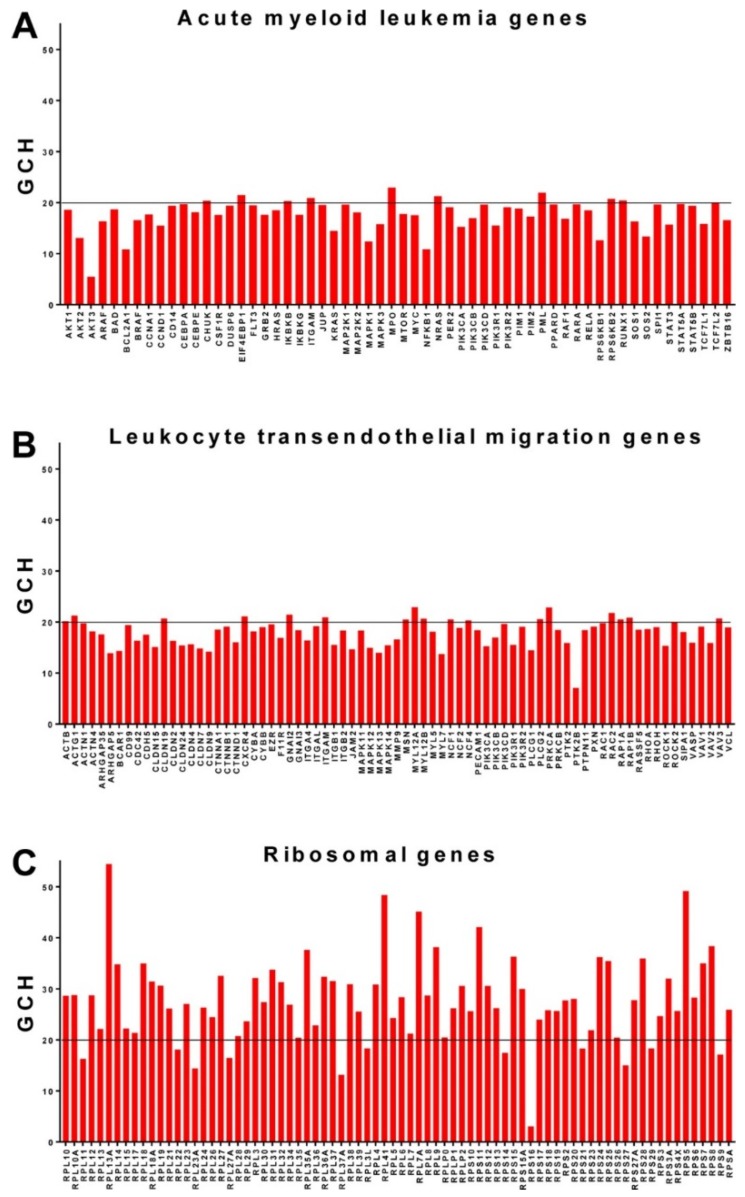
Data from GSE72415: Gene Commanding Height (GCH) scores in the acute promyelocytic leukemia cell line HL-60. (**A**). GCH scores of genes associated with acute myeloid leukemia. (**B**). GHC scores of genes associated with KEGG pathway of leukocyte transendothelial migration. (**C**). GCH scores of the ribosomal genes.

An interesting gene hierarchy was obtained for the HL-60 cells (Figure 5), where most of the ribosomal genes top all KEGG identified genes as associated with acute myeloid leukemia (AML, https://www.kegg.jp/kegg-bin/show_pathway?map=has05221&show_description=show). Thus, the top two AML genes, *MPO* (myeloperoxidase) and *PML* (promyelocytic leukemia) have the GCHs 22.96 and 21.95, below those of the 56th and the 59th ranked ribosomal proteins *RPL29* (23.64) and *RPL13* (22.12). As presented in Figure 5C, genes from both large (RPL) and small (RPS) ribosomal subunits are among the highest ranked genes in the HL-60 cells. According to our results, certain ribosomal genes (*RPL13A*, *RPS5*) are more influential in dictating/preserving the HL-60 phenotype than *RARA* (retinoic acid receptor alpha, GCH = 19.67), the gene whose translocation t (15;17) [54,55] is associated with 98% of acute promyelocytic leukemia cases. Interestingly, *RPL13A* was also found to be the most influential gene in 8505C (anaplastic thyroid cancer) cells and to have a high GCH (63.26) in the BCPAP cells (Table 2 above). For comparison, Figure 5 also presents the GCH scores for the genes associated with the immune system KEGG pathway of leukocyte transendothelial migration (https://www.kegg.jp/kegg-bin/show_pathway?map=hsa04670&show_description=show).

## 4. Discussions

In this report, we propose that the most legitimate targets for the cancer gene therapy are the genes whose highly controlled expressions by the homeostatic mechanisms are the most influential by being correlated with expressions of many other genes. We termed these targets Gene Master Regulators (GMRs), and developed and used the necessary experimental protocol, mathematical algorithm and computer software to identify them from gene expression data. Owing to the astronomic number of possible combinations of risk factors (race, sex, age, medical history, environment, diet, bad habits etc.), each cancer patient has his/her unique gene hierarchy and therefore the GMRs should be identified for each patient separately. Most likely, the gene hierarchy is not rigid but changes in time with the progression of the disease or/and in response to a treatment and environmental changes. Therefore, the gene therapy should be both personalized and timely applied.

Our approach is a significant departure from the identification of aberrantly expressed tumor specific genes that may (or may not) have caused cancerization. Instead, we are looking for the genes whose targeting would selectively kill the cancer cells from the tissue. Such genes have the expression so strongly correlated with that of many other genes that their forced alteration triggers unbearable perturbations of major functional pathways. In tens of previous papers on a large variety of cells and tissues from humans [21,22] and animal (e.g., mouse [10,11,29,38], rat [13,30,39], rabbit [56], dog, chicken embryo [12]) models of human diseases, we proved that major functional pathways remodel in disease, with certain genes being promoted to command positions. We know that a gene is critical for a particular phenotype when its expression is strongly controlled by the homeostatic mechanisms, making it resistant to the small environmental fluctuations and thus very stably expressed across biological replicas.

The GMR approach was applied to our microarray data on five standard (anaplastic thyroid, papillary thyroid, prostate androgen-sensitive and insensitive, and blood) cancer cell lines and ten profiled regions from surgically removed CCRCC (4), prostate (4) papillary thyroid (2) cancers. In each tumor sample, the fully profiled transcriptome(s) of the cancer nodule(s) was(were) compared to that of the cancer-free resection margins. No particular criterion was used in selecting the tumors excepting their availability and to provide enough diversity for the GMR proof of concept. At this stage of the research, the types of cancer are less important. Although the tested population was very small, increasing its size most likely would not provide common targets for different patients but will increase the confidence in the approach. GCH analysis is highly sensitive and reveals that, although similar, humans are not identical. Nor are the gene hierarchies, even in the normal regions or in cancer nodules with the same Gleason score (as in Table 3). Anyhow, collaborative experiments with medical centers are under way to test our approach on several other cancers.

Even though we did our best to select as histo-pathologically homogeneous as possible the regions to profile, the samples were still heterogeneous and cells of other than the dominant phenotype (normal or cancer) affected the results. However, our manuscript does not intend to provide definite results for kidney, papillary and anaplastic thyroid, androgen-sensitive and androgen-insensitive prostate or blood cancers, but to show a way to identify potential targets for gene therapy of any cancer type. Nonetheless, next round of experiments will include separation of the tumor cells into distinct phenotypes with our Sony SH8005 sorter and then profile separately their transcriptomes.

The present studies confirmed that the GMRs may differ even for patients with the same form of cancer (like in the above two prostate cases and in comparisons with corresponding cell lines), justifying the necessity of a personalized approach of cancer gene therapy. We found that the GMRs can be located in any chromosome, that their transcripts can be both coding and non-coding RNAs and that the encoded proteins may be involved in a wide diversity of biological processes. Although we don’t have yet the experimental evidence, most likely the gene hierarchy changes in time, so that the GMRs should be targeted as soon as possible after their identification.

Importantly, we found that the cancer nuclei and the surrounding normal tissues are governed by different GMRs and that manipulation of the expression of a gene has consequences in line with its Gene Commanding Height. Based on these findings, we provide the hypothesis that **silencing the GMR (using CRISPR or shRNA) may selectively kill the cancer cells with little effect on the normal cells of the tissue**. However, we generally we found the GMR to be well above the rest of the genes, with the most notable exception in this report being *WFDC3* (GCH = 173.58) in LNCaP cells (next gene is RPL31 at 39.11). Therefore, this hypothesis would work much better for cases where the GMR has a significantly dominant GCH over the other genes in the cancer nucleus and a very low GCH in the normal cells of the tissue. If the GMR transcript is coding, then its forced alteration affects directly the encoded protein, although coding or non-coding, through the strong expression coordination and manipulation of the GMR should affect the levels of numerous proteins involved in major functional pathways.

At this moment, we do not know whether targeting cancer GMRs may improve the results of the chemo- or radiation therapy, but combining gene manipulation with traditional treatment (as suggested by one of the reviewers) appears to be a very interesting idea.

## Figures and Tables

**Table 1:** Expression data from GSE72304, [21].

GENE	DESCRIPTION	CHR	CTR	PTA	PTB	MET
*DAPK3*	death-associated protein kinase 3	19	**30.31**	4.73	1.15	2.52
*PMPCA*	peptidase (mitochondrial processing) alpha	9	**28.35**	6.82	3.24	4.26
*COA1*	cytochrome c oxidase assembly factor 1 homolog	7	**22.40**	4.83	3.94	1.42
*FAM208A*	family with sequence similarity 208, member A	3	3.08	**63.97**	1.59	5.40
*BCR*	breakpoint cluster region	22	1.15	**57.43**	1.14	1.22
*C2orf81*	chromosome 2 open reading frame 81	2	2.24	**51.24**	3.19	1.84
*FAM27C*	family with sequence similarity 27, member C	9	1.75	6.03	**57.19**	3.73
*GTPBP3*	GTP binding protein 3 (mitochondrial)	19	2.07	29.80	**40.06**	14.01
*MIR1915HG*	MIR1915 host gene	10	2.57	5.55	**31.14**	4.06
*ALG13*	ALG13, UDP-N-acetylglucosaminyltransferase subunit	X	3.64	9.97	2.12	**82.95**
*NUDT18*	nudix (nucleoside diphosphate linked moiety X)-type motif 18	8	1.64	2.69	1.89	**48.40**
*RAD54B*	RAD54 homolog B	8	0.96	6.10	4.09	**40.02**

Gene Commanding Heights (GCH) of the top three genes (bold, grey background) in the two primary tumor (PTA, PTB) regions from the right kidney and chest wall (MET) of a patient with metastatic clear cell renal cell carcinoma (CCRCC) and their GCH scores in the other regions from the analyzed CCRCC sample. CHR = chromosomal location. Note that, although different from one cancer region to the other, the top genes of cancer nuclei (PTA, PTB and MET) have substantially lower GCH scores in the control (normal, CTR) tissue and that the top genes of CTR region have lower GCH scores in the PTA, PTB and MET regions.

**Table 2:** Expression data from GSE97001, GSE97002, [22].

GENE	DESCRIPTION	CHR	NORM	PAP-C	BCPAP	8505C
*RASD1*	RAS, dexamethasone-induced 1	17	**41.51**	4.50	5.70	7.31
*POTEF*	POTE ankyrin domain family, member F	2	**31.17**	8.50	6.90	6.36
*RCN2*	reticulocalbin 2, EF-hand calcium binding domain	15	**31.09**	5.53	7.99	10.38
*SPINT2*	serine peptidase inhibitor, Kunitz type, 2	19	1.93	54.97	18.83	5.88
*RPAP3*	RNA polymerase II associated protein 3	12	5.33	51.74	3.25	12.69
*BZW1*	basic leucine zipper and W2 domains 1	2	2.67	44.32	12.77	26.73
*RPF1*	ribosome production factor 1 homolog	1	8.36	2.22	**135.50**	22.11
*TIMP2*	TIMP metallopeptidase inhibitor 2	17	2.68	6.36	**110.45**	18.04
*ECT2*	epithelial cell transforming 2	3	6.93	8.16	**100.98**	28.15
*SENP5*	SUMO1/sentrin specific peptidase 5	3	9.93	6.32	**100.37**	13.71
*RPL13A*	ribosomal protein L13a	19	13.16	8.73	63.26	**83.02**
*ALDOA*	aldolase A, fructose-bisphosphate	16	7.00	28.05	2.59	**67.30**
*TIPIN*	TIMELESS interacting protein	15	3.11	9.15	37.04	**56.85**

Gene Commanding Heights of the top genes (bold, grey background) in normal (NORM) and cancer (PAP-C) regions of the unilateral tumor removed from a 33-year-old Asian female, with papillary thyroid cancer (pathological stage pT3NOMx). For comparison, we present also the results for the standard BCPAP (papillary) and 8505C (anaplastic) human thyroid cancer cell lines. Owing to their close (and over 100) GCHs, four instead of three genes are listed for the BCPAP cells. Note the differences in GCH scores between NORM and PAP-C. Note also the differences between the GCH scores of genes in the surgically removed carcinoma and the BCPAP cell line, even both are reported as papillary thyroid cancers.

**Table 3:** Data from GSE133891 and GSE133906.

GENE	DESCRIPTION	CHR	NORM1	CANCER1	NORM2	CANCER2	LNCaP	DU145
*TOR1A*	torsin family 1, member A	9	**84.24**	1.91	3.27	10.94	6.57	16.47
*MRPS12*	mitochondrial ribosomal protein S12	19	**80.71**	4.09	3.50	3.04	12.16	15.71
*GTF2H1*	general transcription factor IIH, polypeptide 1	11	**42.66**	5.71	5.27	5.83	4.34	17.34
*BAIAP2L1*	BAI1-associated protein 2-like 1	7	2.06	**49.38**	0.86	2.56	3.72	15.95
*FAM71E1*	family with sequence similarity 71, member E1	19	0.93	**48.21**	1.08	4.49	3.59	16.26
*MAP6D1*	MAP6 domain containing 1	3	1.29	**45.26**	1.34	2.05	7.50	16.61
*SFR1*	SWI5-dependent recombination repair 1	10	2.66	1.36	**40.10**	4.64	5.01	17.10
*EDF1*	endothelial differentiation-related factor 1	9	2.52	1.86	**29.51**	5.75	5.18	17.25
*RHOD*	ras homolog family member D	11	1.25	1.15	**27.90**	2.50	3.92	14.89
*LOC145474*	uncharacterized long non-coding RNA	14	2.23	1.64	1.16	**126.75**	1.01	12.33
*PRRG1*	proline rich Gla (G-carboxyglutamic acid) 1	X	N/A	N/A	1.82	**87.53**	5.10	14.33
*ASAP3*	ArfGAP with SH3 domain, ankyrin repeat and PH domain 3	1	1.19	1.73	1.97	**76.23**	4.15	16.28
*WFDC3*	WAP four-disulfide core domain 3	20	3.57	1.90	1.33	11.61	173.58	15.89
*RPL31*	60S ribosomal protein L31	2	1.30	0.94	2.19	8.25	39.11	18.16
*ALX4*	ALX homeobox 4	11	N/A	N/A	N/A	6.32	35.18	18.16
*VIM*	vimentin	10	1.28	1.97	N/A	2.85	3.51	33.95
*POTEM*	POTE ankyrin domain family, member M	14	1.18	2.40	0.54	4.18	2.51	33.25
*EXOC5*	exocyst complex component 5	14	1.32	1.32	1.03	5.28	2.75	32.19

Gene Commanding Heights of the top three genes (bold, grey background) in normal (NORM) and cancerous (CANCER) regions of surgically removed prostate tumors from a 65y old black male and of a 47y old white male, both with prostatic adenocarcinoma (Gleason score 4 + 5 = 9/10) and negative for adenocarcinoma resection margins. Note the differences in GCH scores between NORMAL and CANCER. Note that the two men have different GMRs in both normal and cancer regions. For comparison, we show also the GCHs of the same genes (and of their own GMRs) for two standard prostate cancer cell lines: the androgen-sensitive LNCaP and the hormone insensitive DU145. Note also the large GCH gap between the first two genes (*WFDC3* and *RPL31*) in the LNCaP cells.
